# Momelotinib therapy for myelofibrosis: a 7-year follow-up

**DOI:** 10.1038/s41408-018-0067-6

**Published:** 2018-03-07

**Authors:** Ayalew Tefferi, Daniela Barraco, Terra L. Lasho, Sahrish Shah, Kebede H. Begna, Aref Al-Kali, William J. Hogan, Mark R. Litzow, Curtis A. Hanson, Rhett P. Ketterling, Naseema Gangat, Animesh Pardanani

**Affiliations:** 1 Division of Hematology, Department of Internal Medicine, Mayo Clinic, Rochester, MN USA; 2 Division of Hematopathology, Department of Laboratory Medicine, Mayo Clinic, Rochester, MN USA; 3 Division of Cytogenetics, Department of Laboratory Medicine, Mayo Clinic, Rochester, MN USA

## Abstract

One-hundred Mayo Clinic patients with high/intermediate-risk myelofibrosis (MF) received momelotinib (MMB; JAK1/2 inhibitor) between 2009 and 2010, as part of a phase 1/2 trial (NCT00935987); 73% harbored *JAK2* mutations, 16% *CALR*, 7% *MPL*, 44% *ASXL1*, and 18% *SRSF2*. As of July 2017, MMB was discontinued in 91% of the patients, after a median treatment duration of 1.4 years. Grade 3/4 toxicity included thrombocytopenia (34%) and liver/pancreatic test abnormalities (<10%); grade 1/2 peripheral neuropathy occurred in 47%. Clinical improvement (CI) occurred in 57% of patients, including 44% anemia and 43% spleen response. CI was more likely to occur in *ASXL1*-unmutated patients (66% vs 44%) and in those with <2% circulating blasts (66% vs 42%). Response was more durable in the presence of *CALR* type 1/like and absence of very high-risk karyotype. In multivariable analysis, absence of *CALR* type 1/like (HR 3.0; 95% CI 1.2–7.6) and presence of *ASXL1* (HR 1.9; 95% CI 1.1–3.2) or *SRSF2* (HR 2.4, 95% CI 1.3–4.5) mutations adversely affected survival. *SRSF2* mutations (HR 4.7, 95% CI 1.3–16.9), very high-risk karyotype (HR 7.9, 95% CI 1.9–32.1), and circulating blasts ≥2% (HR 3.9, 95% CI 1.4–11.0) predicted leukemic transformation. Post-MMB survival (median 3.2 years) was not significantly different than that of a risk-matched MF cohort not receiving MMB.

## Introduction

Momelotinib (MMB; GS-0387; CYT387) is a JAK1 and JAK2 inhibitor, with therapeutic activity in myelofibrosis (MF), in humans as well as in mice^1–4^. In a phase 1/2 study of MMB in patients with MF^1^, maximum tolerated dose was established at 300 mg/day and dose-limiting toxicity included grade 3 headache and hyperlipasemia. The particular phase 1/2 study included 166 patients treated at either 150 or 300 mg once-daily or 150 mg twice-daily for 9 months;^5^ study patients were enrolled between November 2009 and August 2011 and 165 patients received at least one dose of study drug. The particular study included 14% of patients previously exposed to another JAK2 inhibitor therapy. After median (range) treatment duration of 15.3 months (0.1–48.8), the overall response rate in the 166 patients multicenter study was 58% and included no complete remissions and only 1 partial remission and the drug did not affect the mutant *JAK2* allele burden; anemia response was 59% and red cell transfusion-independency was achieved by 75% whereas palpable spleen response was reported at 40%. In terms of treatment-emergent drug toxicity, the most common grade 1/2 adverse events (AEs) included diarrhea, peripheral neuropathy and thrombocytopenia, and first-dose effect of hypotension, dizziness, nausea, headache, and flushing. The current study is limited to the subset of 100 patients treated with MMB at the Mayo Clinic; we provide a 7-year follow-up of patient data, which focuses on overall and leukemia-free survival, as well as the impact of driver and other mutations on response rates and overall, leukemia-free and relapse-free survival.

## Methods

The current study was approved by the Mayo Clinic institutional review board and all patients provided informed written consent for clinical trial participation, study sample collection as well as permission for its use in research. Patient eligibility criteria, study design, treatment plan, study test schedule, and other protocol details have previously been reported^1^, and will not be reiterated here. The study population for the current study constitutes part of a larger phase 1/2 clinical trial (CCL09101; NCT00935987) using momelotinib for the treatment of MF; the results of which were recently communicated^5^. Toxicity was graded by the National Cancer Institute Common Terminology Criteria for Adverse Events (CTCAE) version 4.0. Baseline transfusion status, anemia, and spleen responses were all defined according to the 2006 international working group for MPN research and treatment (IWG-MRT criteria)^6^. Cytogenetic analysis and reporting was done according to the International System for Human Cytogenetic Nomenclature and assignment as “unfavorable karyotype” was according to the previously established criteria^7^. Targeted next-generation sequencing was used to screen for prognostically relevant mutations^8,9^. Information on survival and leukemic transformation was updated in August 2017.

Statistical analyses considered clinical and laboratory parameters obtained at the time of MMB study entry. All statistical analyses considered clinical and laboratory parameters were obtained at the time of MMB study entry. Differences in the distribution of continuous variables between categories were analyzed by either Mann–Whitney or Kruskal–Wallis test. Patient groups with nominal variables were compared by chi-square test. Survival analysis was considered from the date of study entry to the date of death (uncensored) or last contact (censored). Patients receiving allogeneic stem cell transplant (ASCT) were censored at the time of their transplant for survival analysis. Leukemia-free survival calculations considered the transformation event as the uncensored variable. Survival curves were prepared by the Kaplan–Meier method and compared by the log-rank test. The Cox proportional hazard regression model was used for multivariable analysis. *p-*Values less than 0.05 were considered significant. All analyses were conducted using the Stat View (SAS Institute, Cary, NC, USA). In order to obtain preliminary information regarding the impact of MMB therapy on survival, we recruited a retrospective cohort of JAK inhibitor treatment-naïve patients, from our institutional databases, matched for their dynamic international prognostic scoring system (DIPSS-plus) risk status^10^. All data were analyzed by the authors of the current document, without any influence from the sponsor.

## Results

### Patient characteristics at the time of MMB study entry

The current study involves 100 MMB-treated patients (median age 66 years; 58% males) from the Mayo Clinic who were enrolled between 20 November 2009 and 10 November 2010; 64 patients had primary, 22 post-polycythemia vera, and 14 post-essential thrombocythemia MF; 73 (73%) harbored *JAK2* mutations, 16 (16%) *CALR*, 7 *MPL*, and 4 were “triple-negative”; among the 16 *CALR*-mutated cases, 13 were type 1/like. DIPSS-plus^10^risk distribution was 63% high, 36% intermediate-2, and 1% intermediate-1; 49% of the patients displayed red cell transfusion need, 58% constitutional symptoms, 87% palpable splenomegaly >5 cm, and 50% abnormal karyotype; 94 patients were screened for *ASXL1* mutations with 41 (44%) mutated and 78 for *SRSF2* mutations with 14 (18%) mutated; 21 (21%) patients were previously treated with another JAK inhibitor.

### Adverse events

As of July 2017, MMB treatment has been discontinued in 91 (91%) patients, after a median treatment duration of 1.4 years (range 0.02–6.2); median duration of treatment in the nine (9%) patients still receiving the drug was 6.7 years (range 6.3–7.2). The most frequent reason for treatment discontinuation was suboptimal response or progressive disease (59%), including on-study leukemic transformation (3 patients); other reasons included side effects (15%) including peripheral neuropathy (7 patients), on-study deaths (15 patients), and development of secondary malignancies (3 patients). “MMB-related” grade 3 or 4 AEs included thrombocytopenia (34%), neutropenia (9%), anemia (5%), increased serum lipase (7%), alanine aminotransferase (ALT) (4%), aspartate aminotransferase (AST) (2%), alkaline phosphatase (2%) levels, and headaches (2%). In addition, noteworthy grade 1 or 2 AEs included peripheral neuropathy 47%, increased serum lipase (14%), amylase (17%), bilirubin (13%), AST (21%), ALT (19%) levels, increased activated partial thromboplastin time (APTT) (17%), headaches (13%), dizziness (22%), nausea (23%), and diarrhea (20%). Most of the AEs, except peripheral neuropathy, were reversible.

### Treatment efficacy and predictors of response and relapse-free survival

Clinical improvement (CI) was achieved by 57 (57%) patients and included anemia response in 44%, and spleen response in 43% of informative cases. Response in both anemia and spleen was recorded in 12 patients while 27 and 18 patients experienced “spleen only” or “anemia only” response. Fifty-one percent of transfusion-dependent patients became transfusion independent. The majority of patients also had marked improvement in their symptoms. Forty-nine (86%) of the 57 patients with CI have since discontinued treatment, after a median treatment duration of 2.3 years. CI was more likely to occur in *ASXL1*-unmutated (66%) vs mutated (44%) cases (*p* = 0.03) and in patients with circulating blast count <2% (66% vs 42%; *p* = 0.02); CI was not influenced by driver mutational status (*p* = 0.34), DIPSS-plus risk (*p* = 0.97), *SRSF2* mutations (*p* = 0.51), abnormal (*p* = 0.84), or unfavorable (*p* = 0.36) karyotype, prior JAK2 inhibitor therapy (*p* = 0.63), leukocyte count (*p* = 0.17), platelet count (*p* = 0.5), or spleen size (*p* = 0.1). Durability of response was assessed by relapse-free survival, which was adversely affected by absence of type 1/like *CALR* (HR 2.9; 95% CI 1.1–7.3) or presence of very high-risk karyotype (HR 3.5; 95% CI 1.2–10.7).

### Overall and leukemia-free survival analysis

To date, 73 (73%) deaths, occurring at a median of 2.5 years (range 0.06–6.9), and 15 (15%) leukemic transformations, occurring at a median of 3.6 years (range 0.12–7.2), have been recorded. Twenty-seven (27%) patients are currently alive and followed for a median of 6.6 years (range 5.5–7.2) from the time of study entry; among them, eight have received allogeneic stem cell transplant (ASCT) and were censored at the time of the procedure for survival analysis. Median survival from the time of study entry was 3.2 years with 5-year survival of 30%.

In univariate analysis, *ASXL1* and *SRSF2* mutations, absence of *CALR* type 1/like, unfavorable karyotype, circulating blasts ≥2%, older age, and the failure to achieve CI were all associated with inferior post-MMB survival (*p* < 0.05 in all instances). In multivariable analysis not including response status, absence of *CALR* type 1/like (HR 3.0, 95% CI 1.2–7.6) and presence of *ASXL1* (HR 1.9, 95% CI 1.1–3.2) and *SRSF2* (HR 2.4, 95% CI 1.3–4.5) mutations sustained their significance, along with age (*p* = 0.006). Furthermore, the genetic markers remained significant even when CI status was included in the multivariable model: HRs (95% CI) were 2.4 (1.4–4.2) for *ASXL1*/*SRSF2* mutations, 3.0 (1.2–7.7) for absence of *CALR* type 1/like, and 0.37 (0.2–0.6) for CI. In both univariate and multivariable analysis, leukemia-free survival was adversely affected by *SRSF2* mutations (HR 4.7, 95% CI 1.3–16.9), very high-risk karyotype (HR 7.9, 95% CI 1.9–32.1), and circulating blasts ≥2% (HR 3.9, 95% CI 1.4–11.0).

**Fig. 1:**
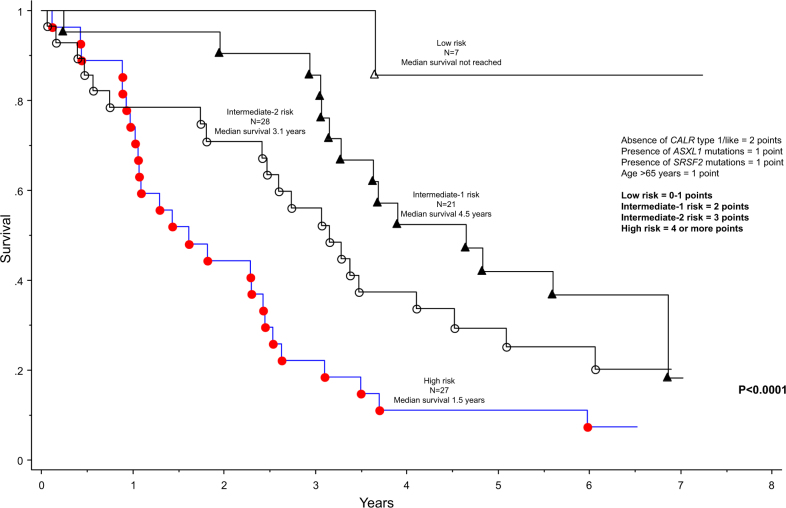
Survival of 83 molecularly annotated patients with myelofibrosis from the time of momelotinib study entry to the last follow-up or death, and stratified by age and mutation profile

**Fig. 2:**
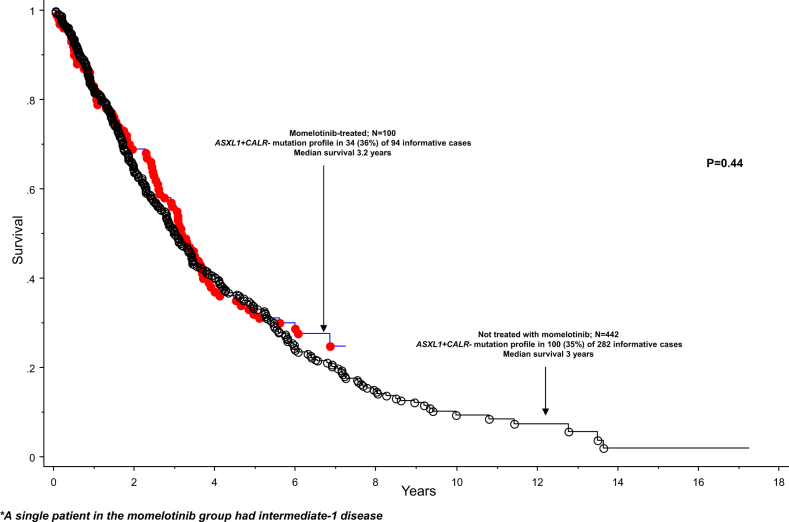
Comparison of survival data between 100 patients receiving and 442 patients not receiving momelotinib treatment; the two populations consisted of DIPSS-plus high or intermediate-2 risk patients only with the exception of a single momelotinib-treated patient who was intermediate-one risk

Post-MMB survival was effectively predicted by an HR-weighted scoring of driver mutational status, presence or absence of *ASXL1* and *SRSF2* mutations, and age (Fig. 1). Comparison of MMB-treated patients with a risk-matched MF cohort not receiving treatment with MMB did not disclose significant difference in survival data (Fig. 2).

## Discussion

It is now well-established that momelotinib treatment in MF provides relief from symptomatic splenomegaly and constitutional symptoms, and unlike most other JAK2 inhibitors, also improves anemia in a substantial fraction of patients^5^. The latter effect might be related to the drug’s inhibitory activity on ALK2-mediated hepcidin expression^11^. Side effects of MMB, including thrombocytopenia and increased serum levels of liver and pancreas enzymes, can be monitored closely and might require drug dose adjustments. On the other hand, drug-induced peripheral neuropathy, which occurs in more than a third of the patients, might require earlier treatment discontinuation because of the possibility of irreversible damage^12^. Our long-term experience suggests that MMB is otherwise well tolerated and capable of inducing durable benefit, in a subset of molecularly appropriate patients, and without the unwanted side effect of drug-induced anemia.

The pathway towards regulatory approval of MMB appears to be uncertain, judging from the underwhelming performance of the drug in two recent randomized studies. In JAK2 inhibitor-naïve patients^13^, MMB was compared to ruxolitinib and among patients completing 24 weeks of therapy, spleen response rates were similar (26.5% vs 29%) while anemia response rates were superior with MMB (transfusion-independence rate of 66.5% vs 49.3%) and symptoms response rates with ruxolitinib (42.2% vs 28.4%); grade 3/4 anemia occurred more frequently with ruxolitinib (23% vs 6%) while other AEs were reported to be similar. In the second phase 3 study of patients previously treated with ruxolitinib^14^, MMB was compared to best available therapy (BAT); among patients completing 24 weeks of treatment, spleen responses were not impressive in both arms of the study (6.7% vs 5.8%) while anemia (transfusion-independence rate of 43.3% vs 21.2%) and symptoms response rates (26.2% vs 5.9%) were superior with MMB. Of note, in the latter study^14^, ruxolitinib was included as part of BAT in 88% of the patients.

The palliative benefit of MMB and other JAK2 inhibitors comes at a cost to patients and this should be clearly communicated to them prior to starting treatment^15^. In this regard, the emphasis should be on peripheral neuropathy for MMB and anemia for ruxolitinib. Long-term use of ruxolitinib has also been associated with opportunistic infections, because of the immunosuppressive effects of the drug^16,17^. Patients should not be misled regarding the disease-modifying activity MMB or other JAK2 inhibitors, and there is no consistent evidence that these drugs can reverse MF or induce cytogenetic or molecular remissions; the mechanism of action for this class of drugs has been attributed to non-specific suppression of inflammatory cytokines, which explains their salutary activity in other unrelated conditions^18,19^. The current study underscores the transient nature of JAK2 inhibitor-induced palliation of symptoms in MF, as well as the limited effect on overall survival, which is instead primarily determined by the underlying molecular signature of the disease^20,21^. From a practical standpoint, these observations suggest that MF patients with *SRSF2* or *ASXL1* mutations or very high-risk karyotype might be better served by ASCT sooner than later. The use of JAK inhibitors in such cases is unlikely to modify the poor prognosis imparted by the associated adverse mutations and might compromise the sense of urgency for ASCT.
